# Histology-Stratified Clinical and Prognostic Associations of PDCD-Related Genes in Lung Adenocarcinoma and Lung Squamous Cell Carcinoma

**DOI:** 10.3390/genes17101238

**Published:** 2026-10-08

**Authors:** Seong-Tae Kim, Yunha Lee, Se-Young Yun, Jin-Woo Min, Eun-Chan Jeon, Se-Hyun Kim, Jin-Sub Yoo, Se-Jin Park, Ji-Hwan Jung, Jun-Chae Lee, Jae-Ho Lee

**Affiliations:** 1 Medical Course, School of Medicine, Keimyung University, Daegu 42601, Republic of Korealyh3dland3@gmail.com (Y.L.); seyounghappy@naver.com (S.-Y.Y.); minjinwoof1@daum.net (J.-W.M.); skylar3007@naver.com (S.-H.K.); jinsup55@naver.com (J.-S.Y.); qkrtpwls1226@naver.com (S.-J.P.); chai1703@naver.com (J.-C.L.); 2 Department of Anatomy, School of Medicine, Keimyung University, Daegu 42601, Republic of Korea

**Keywords:** *PDCD*, *AVEN*, lung adenocarcinoma, lung squamous cell carcinoma, overall survival, false discovery rate

## Abstract

Background: The prognostic relevance of PDCD-related genes in non-small cell lung cancer remains uncertain. We examined *PDCD2*, *PDCD4*, *PDCD5*, *PDCD10*, and *AVEN* separately in lung adenocarcinoma (LUAD) and lung squamous cell carcinoma (LUSC). Methods: Matched TCGA expression cohorts comprised 492 LUAD and 484 LUSC tumors. Primary Cox models evaluated continuous expression per standard deviation using separate gene-specific models adjusted for age, sex, smoking history, and pathologic stage. Benjamini–Hochberg correction covered ten gene–histology tests. Proportional hazards, multicollinearity, model specification, and the incremental value of *PDCD4* were assessed. GSE30219 provided 83 LUAD and 61 LUSC patients for unadjusted and clinically adjusted external analyses. Hallmark enrichment was evaluated using legacy RSEM expression and a STAR TPM sensitivity analysis. Results: The primary TCGA models included 469 LUAD patients with 167 deaths and 467 LUSC patients with 205 deaths. *AVEN* was associated with poorer LUAD survival after correction (HR 1.272, 95% CI 1.091–1.483; q = 0.022). *PDCD4* was not significant in the primary LUAD model (HR 0.875, 95% CI 0.749–1.021; q = 0.150). Adding *PDCD4* to clinical variables yielded a C-index increase of 0.014 (bootstrap 95% CI −0.004 to 0.031). No adjusted external association survived correction. *PDCD4*-associated enrichment differed between expression pipelines. Conclusions: *AVEN* showed an exploratory adverse association in TCGA LUAD, but external prognostic replication was not established. These analyses do not support a robust independent prognostic claim for *PDCD4* or the clinical use of any of the five evaluated genes.

## 1. Introduction

Lung cancer remains a leading cause of cancer incidence and mortality worldwide [1]. Lung adenocarcinoma (LUAD) and lung squamous cell carcinoma (LUSC), the two major histological subtypes of non-small cell lung cancer (NSCLC), exhibit distinct molecular characteristics. Comprehensive genomic analyses have demonstrated that LUAD is frequently characterized by alterations involving receptor tyrosine kinase and RAS–RAF signaling pathways, whereas LUSC more commonly exhibits abnormalities involving TP53, CDKN2A, SOX2, PIK3CA, and related pathways [2,3,4]. These molecular differences support histology-stratified investigation of candidate expression markers rather than assuming uniform associations across NSCLC.

Resistance to cell death is a central feature of cancer biology [5]. The genes evaluated in the present study have diverse cellular functions and should not be interpreted as components of a single experimentally established signaling pathway. PDCD2 functions as a chaperone involved in the biogenesis of the small ribosomal subunit [6]. PDCD4 suppresses translation initiation through interaction with eukaryotic initiation factor 4A (eIF4A) [7]. PDCD5 has been implicated in p53 regulation and cellular responses to genotoxic stress [8,9]. PDCD10, also known as CCM3, participates in multiple cellular processes involving cell junctions, proliferation, apoptosis, and vascular homeostasis [10]. AVEN, historically also referred to as PDCD12, interacts with BCL-xL and APAF1 and inhibits caspase activation [11].

Previous studies have investigated individual PDCD-related genes in cancer; however, their clinical and prognostic associations may depend on tumor histology and cellular context. In addition, expression-based prognostic associations derived from retrospective transcriptomic cohorts may be sensitive to expression modeling, covariate selection, multiple testing, and cohort composition.

Therefore, the present study aimed to compare the clinical, molecular, and prognostic associations of *PDCD2*, *PDCD4*, *PDCD5*, *PDCD10*, and *AVEN* between LUAD and LUSC. We analyzed transcriptomic and clinicopathological data from TCGA, evaluated survival using continuous-expression models as the primary approach while retaining median-based analyses as descriptive secondary analyses, and assessed reproducibility in the independent GSE30219 cohort. We additionally incorporated multiple-testing correction, proportional-hazards diagnostics, multicollinearity assessment, nonlinear sensitivity analyses, and evaluation of the incremental prognostic contribution of *PDCD4*. Gene set enrichment analysis (GSEA) was used to explore expression-associated biological programs without inferring direct causal regulation.

## 2. Materials and Methods

### 2.1. TCGA Data Acquisition and Cohort Assembly

Transcriptomic and clinicopathological data for LUAD and LUSC were obtained from TCGA and matched with primary-tumor expression profiles available through UCSC Xena [12]. The original clinical files contained 494 LUAD and 489 LUSC records. One LUAD record corresponded to a non-primary-tumor identifier, one additional LUAD record lacked a complete target-gene profile, and five LUSC records lacked at least one target-gene measurement. The resulting expression cohorts therefore included 492 patients with LUAD and 484 with LUSC. Availability of a positive overall survival time and valid event indicator yielded survival-eligible populations of 490 LUAD and 483 LUSC patients.

The five target genes were *PDCD2*, *PDCD4*, *PDCD5*, *PDCD10*, and *AVEN*. *AVEN* was used consistently as the current gene symbol; its historical designation *PDCD12* was considered synonymous where required for probe annotation.

Primary analyses used the TCGA legacy RNA-seq RSEM normalized-count resource supplied on a log2(normalized count + 1) scale. A separate STAR TPM expression resource was used for GSEA sensitivity analysis. Missing clinical covariates were handled by complete-case analysis without imputation.

**Figure 1:**
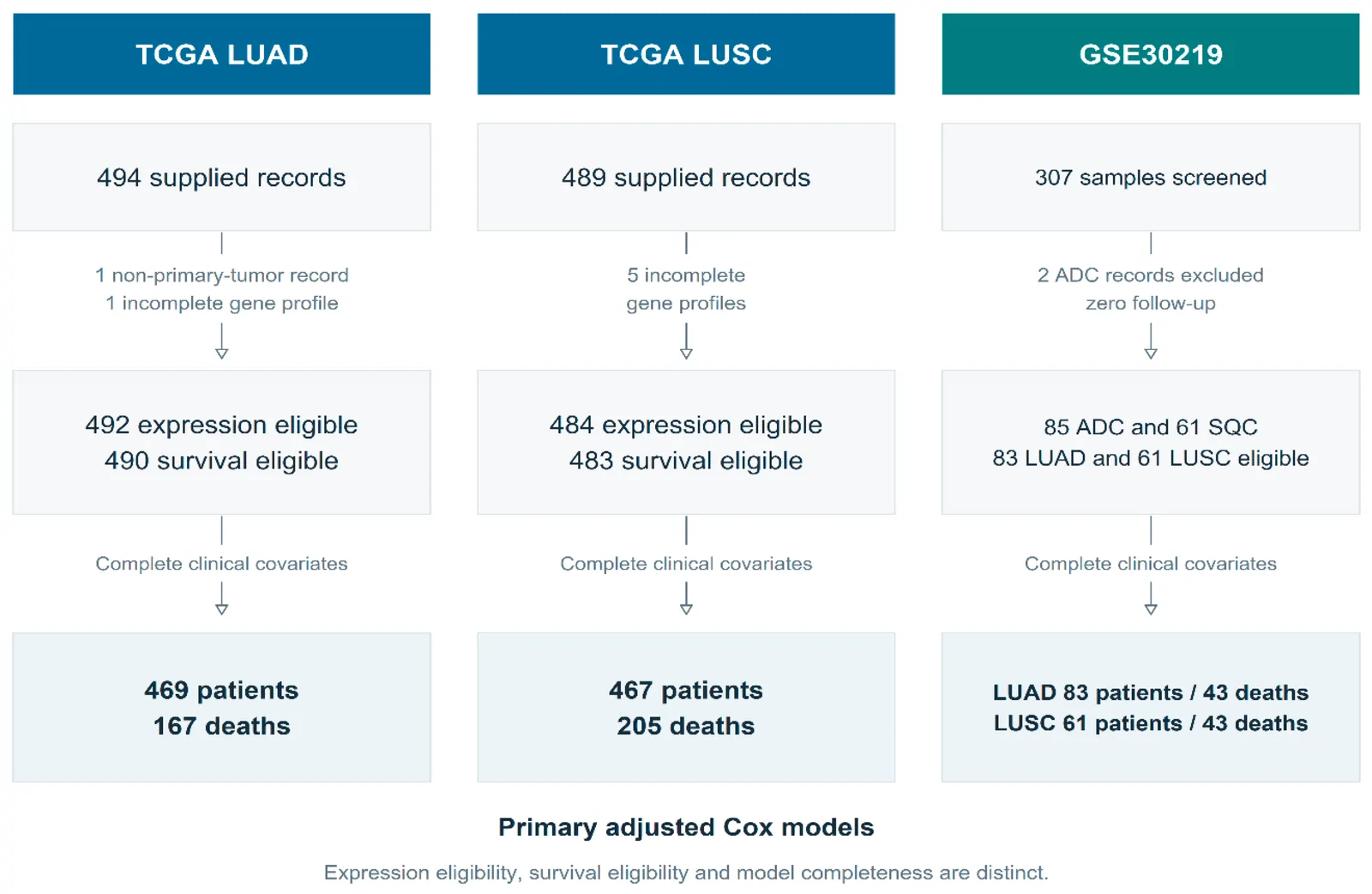
Assembly of the TCGA and GSE30219 analysis populations. Expression eligibility, survival eligibility, and complete-case populations used for the primary adjusted Cox analyses are shown separately. ADC and SQC denote the original GSE30219 adenocarcinoma and squamous carcinoma annotations corresponding to LUAD and LUSC, respectively.

Smoking history was reconstructed from the TCGA clinical field tobacco_smoking_history, with code 1 classified as never smoking and codes 2–5 as ever smoking; other or missing values were treated as unknown. Age was entered into Cox models continuously per 10-year increase, and pathologic stage was modeled categorically with stage I as the reference. EGFR mutation status was examined in sensitivity analyses because mutation information was incomplete. Cohort assembly is summarized in Figure 1.

### 2.2. Clinicopathological and Co-Expression Analyses

For descriptive clinicopathological analyses, patients were classified into high- and low-expression groups using the histology-specific median expression of each gene. Expression above the median was classified as high, whereas expression at or below the median was classified as low. Age was categorized as ≤65 versus >65 years.

Associations between the expression group of each gene and clinicopathological characteristics, including age group, sex, smoking status, pathologic stage, and individual T, N, and M categories, were evaluated using the Pearson’s chi-square test or Fisher’s exact test, as appropriate. Missing values were excluded from the corresponding comparison.

Pearson correlation coefficients were calculated using continuous log2 gene-expression values, and Spearman correlations were additionally examined as sensitivity analyses. Correlations were evaluated among the five target genes and between each target gene and histology-relevant oncogenic-driver transcripts. The LUAD panel included EGFR, KRAS, TP53, ALK, and BRAF, whereas the LUSC panel included TP53, CDKN2A, SOX2, PIK3CA, and NOTCH1.

These analyses were interpreted as transcript co-expression associations and not as evidence of mutation status, physical interaction, regulatory direction, or functional dependence.

Benjamini–Hochberg FDR correction was applied across the prespecified families of clinicopathological and correlation tests.

### 2.3. Overall Survival Analysis and Model Diagnostics

Overall survival was defined from the available TCGA follow-up and death information, with all-cause death treated as the event and patients otherwise censored at the last recorded follow-up.

For the primary survival analysis, expression of each target gene was standardized separately within each histological cohort using the mean and standard deviation of the eligible expression population. Separate Cox proportional hazards models were fitted for each gene. Each primary model included one continuous gene-expression term together with age, sex, corrected smoking history, and categorical pathologic stage. Hazard ratios therefore represent the relative hazard associated with a one-SD increase in log2 gene expression.

The clinically adjusted continuous-expression Cox models described above constituted the primary survival analyses for prognostic inference. All other survival analyses, including unadjusted continuous Cox models, median-dichotomized Cox models, simultaneous five-gene models, alternative stage coding, stage-stratified models, EGFR-adjusted models, nonlinear models, and the incremental assessment of *PDCD4*, were treated as secondary, sensitivity, or exploratory analyses intended to evaluate robustness rather than redefine the primary findings. Median-based Kaplan–Meier curves and two-sided log-rank tests were retained as descriptive secondary analyses; no outcome-optimized expression threshold was selected.

The proportional hazards assumption was formally assessed using scaled Schoenfeld residuals for individual terms and globally according to the approach of Grambsch and Therneau [13]. Stage-stratified models were additionally evaluated when stage-related nonproportionality was detected. Potential nonlinear relationships between continuous gene expression and survival were examined using natural cubic splines with three degrees of freedom and compared with linear models using likelihood-ratio tests.

Multicollinearity in the simultaneous five-gene models was assessed using generalized variance inflation factors and corresponding adjusted variance inflation measures. The number of patients and deaths included in each fitted model was recorded.

Supplementary Figure S4 shows spline-based analyses of potential nonlinear associations between continuous *PDCD4* expression and overall survival.

Supplementary Figure S5 shows representative scaled Schoenfeld residual plots for the *PDCD4* and *AVEN* LUAD models.

### 2.4. Incremental Prognostic Assessment of PDCD4

To evaluate whether *PDCD4* provided prognostic information beyond conventional clinical variables, an exploratory LUAD model containing age, sex, corrected smoking history, and categorical pathologic stage was compared with an otherwise identical model additionally containing continuous *PDCD4* expression.

Harrell’s C-index and a likelihood-ratio test were used to compare the two models. Patient-level bootstrap resampling with 1000 replicates was used to estimate confidence intervals and optimism-corrected C-indices. The bootstrap interval for the difference in apparent C-index was calculated using paired bootstrap differences.

This analysis was considered exploratory and was not interpreted as prospective validation of a clinical prediction model.

### 2.5. External Assessment Using GSE30219

The GSE30219 dataset was obtained from the Gene Expression Omnibus and analyzed using the Affymetrix Human Genome U133 Plus 2.0 platform (GPL570; Affymetrix, Santa Clara, CA, USA) [14]. Among 307 samples, 85 were annotated as adenocarcinoma and 61 as squamous carcinoma. Two adenocarcinoma samples had zero recorded follow-up and were excluded, leaving 83 patients with LUAD and 61 with LUSC.

The retained probes were 204025_s_at for *PDCD2*, 202731_at for *PDCD4*, 219275_at for *PDCD5*, 210907_s_at for *PDCD10*, and 219366_at for *AVEN*. Probe selection was based on expression variability and did not use survival outcomes. Expression values were already log2-scaled and were not transformed again.

Each gene was standardized within histology, and Cox models were fitted both without adjustment and with adjustment for age, sex, T stage, and N stage. T stage was grouped as T1 versus T2–T4, and N stage as N0 versus N1–N3. Smoking history, EGFR mutation status, and directly comparable overall stage were not available, and therefore the external adjusted models did not reproduce the exact TCGA covariate set.

Median-based Kaplan–Meier analyses were retained as secondary analyses using histology-specific medians.

### 2.6. Gene Set Enrichment Analysis (GSEA)

Hallmark GSEA was performed separately within LUAD and LUSC after dividing patients into high- and low-expression groups according to the histology-specific median expression of each target gene [15,16]. Primary GSEA used the matched TCGA legacy RSEM expression resource, whereas STAR TPM data were analyzed as a sensitivity dataset.

GSEA was performed using GSEApy version 1.3.1 [17] and the MSigDB Hallmark collection version 2025.1 [16]. Ranking was based on the signal-to-noise statistic comparing high- and low-expression groups. A weighted enrichment exponent of 1, gene-set size limits of 15–500 genes, 1000 phenotype-label permutations, and a fixed random seed were used. The stratifying gene was excluded from its own ranked input.

Positive normalized enrichment scores (NESs) indicate enrichment in the high-expression group, whereas negative NES values indicate enrichment in the low-expression group. Nominal *p* values, native GSEA FDR q values, family-wise error estimates, matched gene-set sizes, and leading-edge genes were retained. The reported FDR q values were calculated separately within each individual 50-Hallmark-gene-set GSEA run for a given target gene, histological subtype, and expression resource. No additional FDR correction was applied across the complete set of gene-, histology-, and expression-resource-specific GSEA analyses. Accordingly, q < 0.25 was treated as an exploratory within-run enrichment threshold, with q < 0.05 interpreted as stronger within-run evidence.

Because GSEA was not adjusted for tumor purity, immune-cell composition, stromal content, or clinical covariates, enrichment findings were interpreted as bulk-expression associations rather than evidence that the corresponding PDCD-related gene directly regulates a pathway.

### 2.7. Statistical Analysis and Multiple-Testing Correction

All statistical tests were two-sided. For survival analyses, the Benjamini–Hochberg false discovery rate procedure [18] was applied across the ten gene–histology tests comprising five genes evaluated in two histological subtypes. Separate corrections were performed for the defined model families. The same procedure was also applied to the clinicopathological and Pearson correlation test families. Findings with *p* < 0.05 but q ≥ 0.05 were described as nominal associations rather than statistically significant corrected findings. Diagnostic tests and the exploratory *PDCD4* incremental-value comparison were not subjected to multiplicity correction and were interpreted accordingly.

Survival and clinical analyses were performed using R version 4.5.3. GSEA data preparation and enrichment analyses were conducted using Python version 3.12.14 and GSEApy version 1.3.1.

## 3. Results

### 3.1. Cohort Characteristics

The TCGA expression cohorts included 492 patients with LUAD and 484 with LUSC. Survival-eligible subsets included 490 LUAD patients with 178 deaths and 483 LUSC patients with 210 deaths. After complete-case selection for the primary clinical covariates, the adjusted Cox models included 469 LUAD patients with 167 deaths and 467 LUSC patients with 205 deaths.

The external GSE30219 cohort included 83 LUAD patients and 61 LUSC patients, with 43 deaths in each histological subgroup.

Median follow-up by reverse Kaplan–Meier analysis was 2.36 years in TCGA LUAD and 2.99 years in TCGA LUSC, compared with 9.67 and 16.58 years in GSE30219 LUAD and LUSC, respectively. Baseline characteristics and analysis populations are summarized in Table 1.

### 3.2. Clinicopathological and Co-Expression Associations

Median-based clinicopathological analyses identified several nominal associations across the five genes and two histological subtypes. However, after Benjamini–Hochberg correction across the 70 clinicopathological comparisons, only three associations remained statistically significant: *PDCD5* expression group with smoking history in LUAD (*p* = 0.00115, q = 0.039), *PDCD2* expression group with age category in LUSC (*p* = 0.00167, q = 0.039), and *PDCD5* expression group with sex in LUSC (*p* = 0.00067, q = 0.039). Complete counts, denominators, nominal *p* values, and FDR-adjusted q values are provided in Supplementary Table S2.

Co-expression patterns differed between LUAD and LUSC. In LUAD, *PDCD2* showed moderate positive correlations with *PDCD5* and *PDCD10*, whereas *PDCD4* showed comparatively weaker or negative associations with several other target genes. Correlations with oncogenic-driver transcripts were generally small to moderate in magnitude.

The LUAD *PDCD2*–EGFR expression correlation was r = −0.231 (*p* = 2.09 × 10^−7^; q = 9.12 × 10^−7^). Complete Pearson coefficients, *p* values, q values, and corresponding Spearman sensitivity estimates are provided in Supplementary Table S3. Statistically significant correlations, particularly those with small absolute correlation coefficients, were interpreted as co-expression associations rather than evidence of functional interaction or direct regulation.

### 3.3. Primary Continuous-Expression Survival Analysis in TCGA

In the primary multivariable analysis, *AVEN* was the only target gene that remained significant after correction across the ten gene–histology tests. In LUAD, higher *AVEN* expression was associated with poorer overall survival (HR per 1 SD = 1.272, 95% CI = 1.091–1.483; *p* = 0.002; q = 0.022).

*PDCD5* and *PDCD10* showed nominal adverse associations in LUAD, with HRs of 1.186 (95% CI = 1.027–1.370; *p* = 0.020; q = 0.067) and 1.168 (95% CI = 1.006–1.355; *p* = 0.041; q = 0.103), respectively, but neither remained significant after FDR correction.

*PDCD4* showed a favorable point estimate in LUAD but was not significant in the primary model (HR = 0.875, 95% CI = 0.749–1.021; *p* = 0.090; q = 0.150).

**Figure 2:**
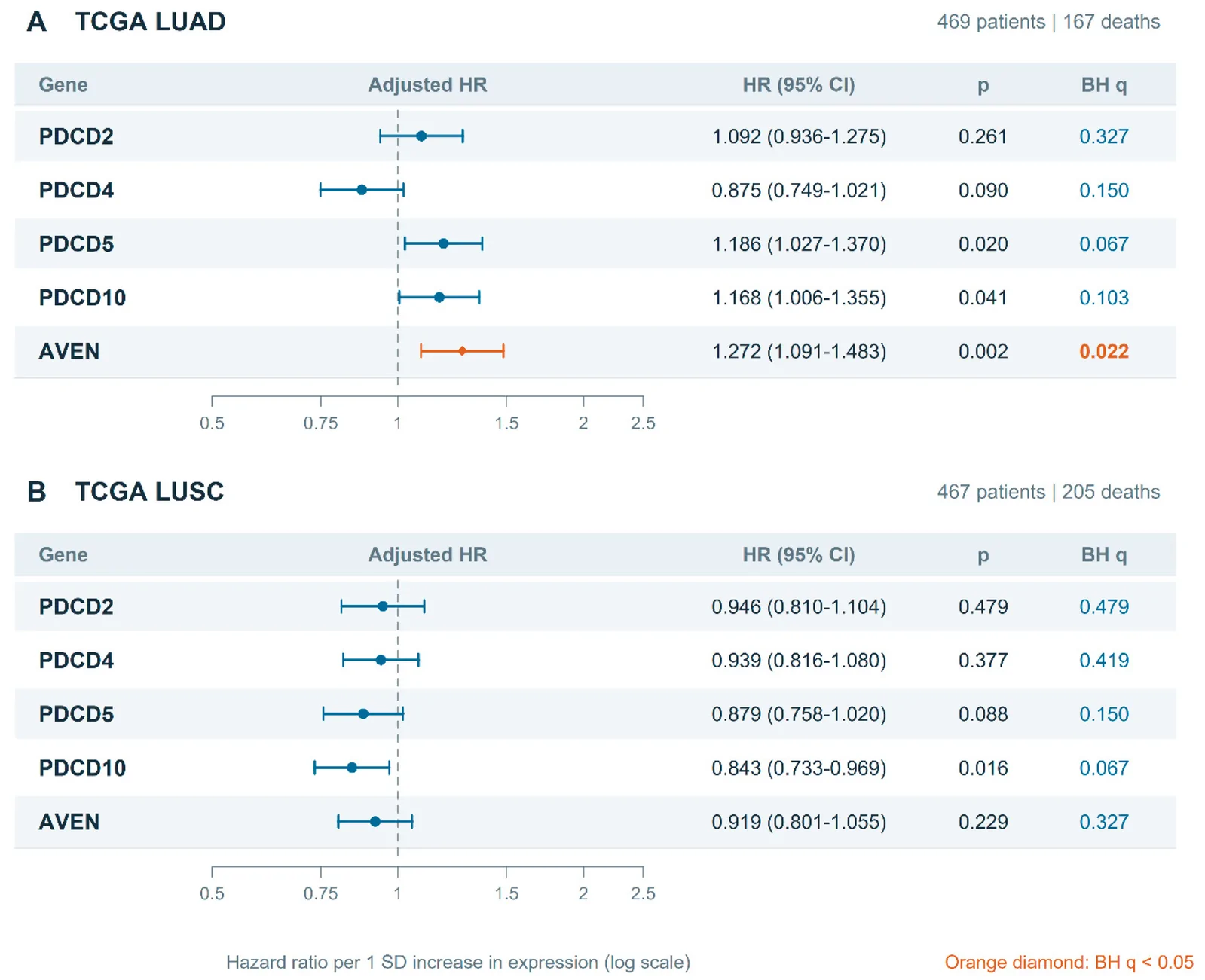
Primary TCGA Cox estimates per one-SD increase in gene expression. Separate models were adjusted for age, sex, corrected smoking history, and categorical pathologic stage. Points indicate hazard ratios and horizontal bars indicate 95% confidence intervals. Panels (**A**,**B**) show LUAD and LUSC, respectively. The orange diamond denotes q < 0.05 after Benjamini–Hochberg correction across the ten gene–histology tests.

In LUSC, *PDCD10* showed a nominal favorable association with survival (HR = 0.843, 95% CI = 0.733–0.969; *p* = 0.016; q = 0.067), but no LUSC gene met the corrected q < 0.05 threshold. The primary Cox estimates are summarized in Table 2 and Figure 2.

### 3.4. Secondary Median-Based Survival Analyses

Kaplan–Meier analyses using histology-specific median expression cutoffs were retained as secondary descriptive analyses.

In LUAD, *PDCD2*, *PDCD4*, *PDCD5*, and *AVEN* showed nominal differences in median-based survival comparisons, whereas *PDCD10* did not. After correction across the ten median-based log-rank tests, *PDCD5* and *AVEN* remained significant in LUAD.

In LUSC, *PDCD10* demonstrated a favorable median-based survival difference and remained significant after correction within this secondary analysis family.

Because these analyses dichotomize continuous expression and may be cutoff-dependent, they were not used as the primary basis for prognostic inference.

Secondary Kaplan–Meier analyses are provided in Supplementary Figure S1.

### 3.5. Model Diagnostics and Sensitivity Analyses

No primary target-gene term showed evidence of violation of the proportional hazards assumption at *p* < 0.05. In representative LUAD models, the Schoenfeld test *p* values were 0.809 for *PDCD4* expression and 0.472 for *AVEN* expression. In contrast, stage terms showed evidence of time-dependent effects in some LUAD models, including *p* = 0.038 in the *PDCD4* model and *p* = 0.049 in the *AVEN* model. Stage-stratified sensitivity models were therefore examined. Importantly, the adverse association of *AVEN* with overall survival in LUAD remained similar in the stage-stratified sensitivity analysis (HR = 1.267, 95% CI = 1.084–1.480; q = 0.029), consistent in direction and magnitude with the primary adjusted analysis.

The maximum target-gene variance inflation factor in the simultaneous five-gene TCGA models was 1.59, providing no evidence of severe linear multicollinearity. However, simultaneous-gene coefficients were interpreted cautiously because they remain conditional on the selected model and complete-case sample.

Nonlinearity analyses did not identify significant departures from linearity after multiple-testing correction. Complete diagnostic results are provided in Supplementary Table S5 and Supplementary Figures S4 and S5.

### 3.6. Incremental Prognostic Information from PDCD4

In the 469-patient LUAD complete-case subset, the clinical model containing age, sex, corrected smoking history, and pathologic stage had an apparent C-index of 0.667. Addition of continuous *PDCD4* increased the apparent C-index to 0.680.

The apparent C-index difference was 0.014, but the paired bootstrap 95% CI ranged from −0.004 to 0.031 and therefore included zero. Optimism-corrected C-indices were 0.651 for the clinical model and 0.663 after addition of *PDCD4*.

The nested likelihood-ratio comparison was also not significant (χ^2^ = 2.868, df = 1; *p* = 0.090). The incremental-performance results are summarized in Table 3.

### 3.7. External Assessment in GSE30219

No adjusted association in the GSE30219 cohort remained significant after FDR correction across the ten gene–histology tests.

In LUAD, PDCD2 and PDCD10 showed nominal adverse adjusted associations, with HRs of 1.481 (95% CI = 1.023–2.145; *p* = 0.037; q = 0.187) and 1.518 (95% CI = 1.105–2.087; *p* = 0.010; q = 0.100), respectively.

The AVEN point estimate was also adverse in LUAD (HR = 1.356, 95% CI = 0.961–1.912), but the association was not statistically significant after adjustment (*p* = 0.083; q = 0.276).

PDCD4 was not significant in LUAD (HR = 0.837, 95% CI = 0.637–1.098; *p* = 0.199; q = 0.332).

**Figure 3:**
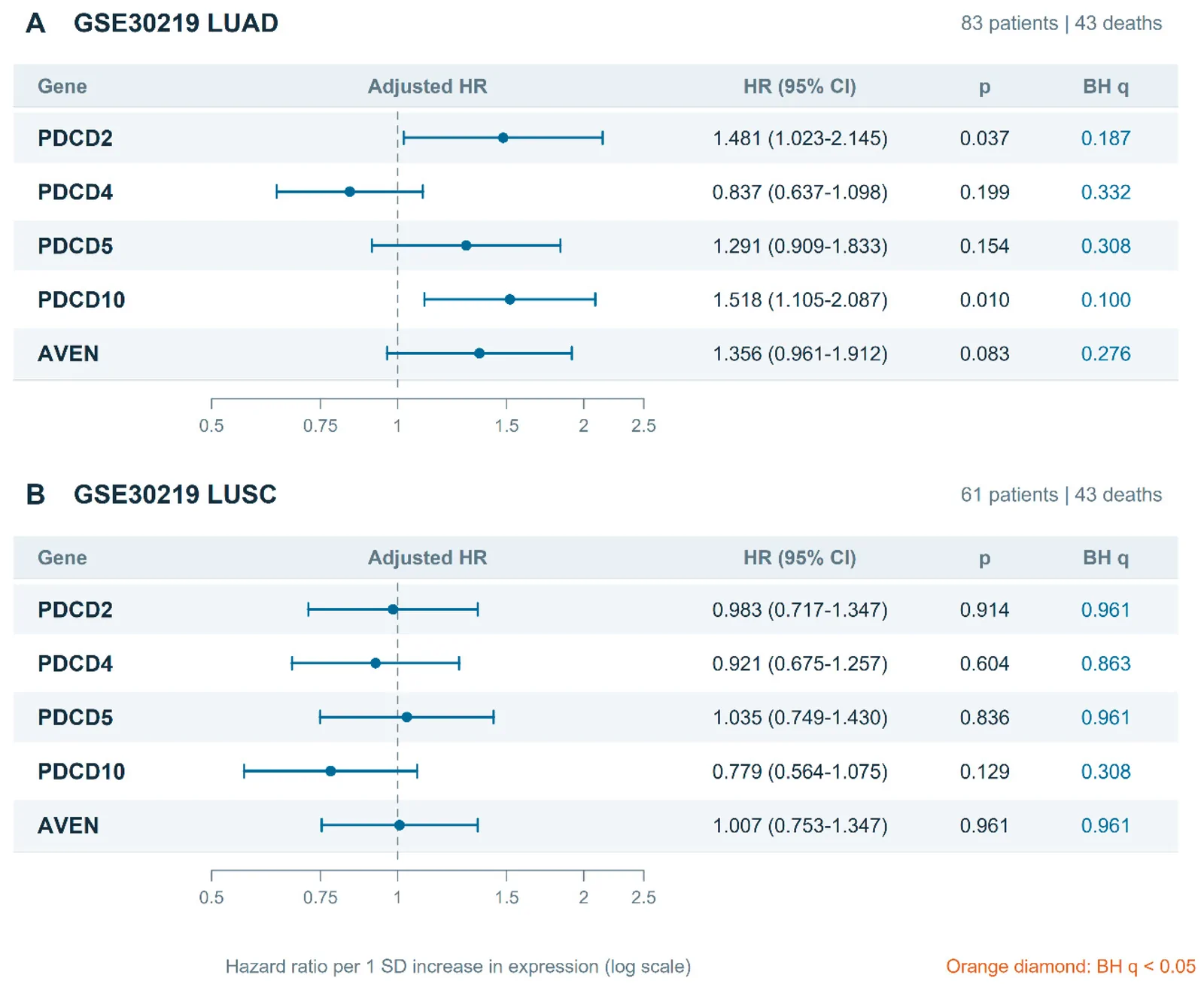
Adjusted continuous-expression Cox analyses in GSE30219. Models were adjusted for age, sex, grouped T stage, and grouped N stage. Panels (**A**,**B**) show LUAD and LUSC, respectively. No association remained significant after Benjamini–Hochberg correction across the ten gene–histology tests. Secondary median-based Kaplan–Meier analyses are shown in Supplementary Figure S2.

In LUSC, none of the five genes showed a significant adjusted association. External survival estimates are summarized in Table 4 and Figure 3.

### 3.8. Exploratory Hallmark Pathway Associations

Hallmark GSEA demonstrated gene-, histology-, and expression-resource-dependent enrichment patterns.

Using the legacy RSEM resource, low PDCD4 expression in LUAD was associated with enrichment of glycolysis (NES = −2.050; q = 0.011), DNA repair (NES = −2.103; q = 0.014), and mTORC1 signaling (NES = −1.891; q = 0.033). In LUSC, low PDCD4 expression was associated with several proliferative and metabolic pathways, including G2M checkpoint, E2F targets, DNA repair, mTORC1 signaling, MYC targets, and glycolysis.

**Figure 4:**
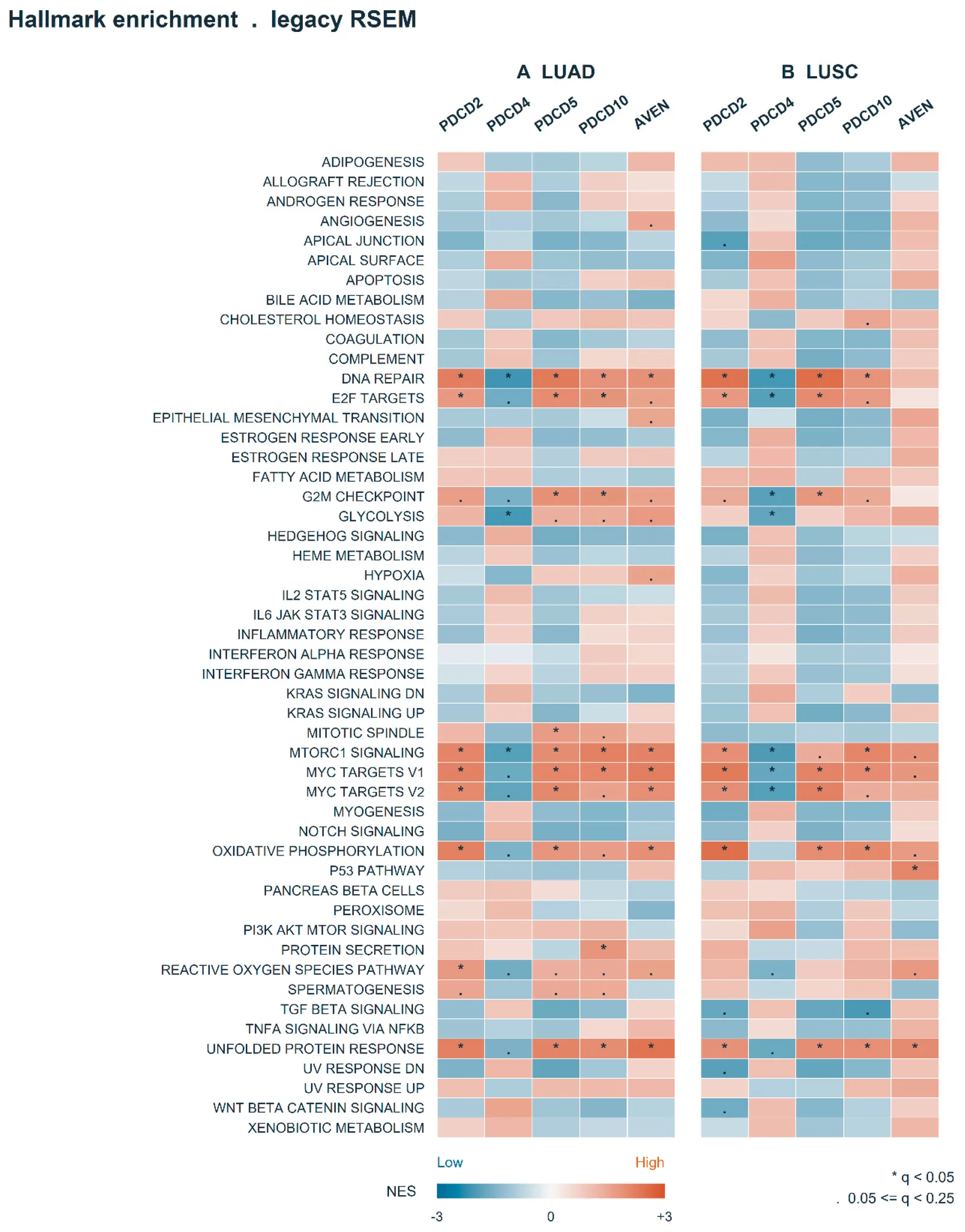
Hallmark gene set enrichment analysis using the TCGA legacy RSEM expression resource. Orange indicates positive enrichment in the high-expression group and blue indicates negative enrichment in the low-expression group. Stars indicate native GSEA FDR q < 0.05, whereas dots indicate 0.05 ≤ q < 0.25. FDR q values were calculated separately within each individual 50-pathway GSEA run. Panels (**A**,**B**) show LUAD and LUSC, respectively. The NES color scale is clipped at −3 and +3 for visualization; complete values are provided in Supplementary Table S7. These analyses were not adjusted for clinical or cell-composition confounding.

In LUAD, high AVEN expression was associated with several proliferative and metabolic Hallmark pathways. These included unfolded protein response (NES = 2.423; q = 0.0013), MYC targets V1 (NES = 2.235; q = 0.0026), and mTORC1 signaling (NES = 2.142; q = 0.0061), together with MYC targets V2, DNA repair, and oxidative phosphorylation. The legacy RSEM Hallmark enrichment results are shown in Figure 4.

## 4. Discussion

The present study evaluated the clinical, molecular, and prognostic associations of five PDCD-related genes using a common analytical framework in LUAD and LUSC. Continuous-expression survival models, multiple-testing correction, formal model diagnostics, and external assessment were incorporated to distinguish robust associations from findings that depended on analytical specification.

After adjustment for established clinical covariates and correction across the ten gene–histology tests, AVEN was the only target gene that remained associated with overall survival, with higher expression associated with poorer survival in TCGA LUAD. PDCD4 showed a favorable direction of association but was not statistically significant in the primary continuous-expression model and did not demonstrate clear incremental prognostic value beyond conventional clinical factors. Furthermore, no adjusted external association remained significant after multiplicity correction in GSE30219. Collectively, these findings support a hypothesis-generating interpretation rather than classification of any of the evaluated genes as a validated prognostic biomarker.

### 4.1. PDCD4

PDCD4 has well-established tumor-suppressive functions, including inhibition of translation initiation through interaction with eIF4A, and reduced PDCD4 expression has previously been associated with tumor progression in lung cancer [7,19]. These biological observations provide a plausible context for the favorable survival pattern observed in the secondary median-based LUAD analysis.

However, the prognostic association of PDCD4 was not robust across analytical specifications. Continuous PDCD4 expression was not significant in the primary adjusted LUAD model after multiple-testing correction, and addition of PDCD4 to clinical variables resulted in only a small and statistically uncertain increase in C-index. Furthermore, the PDCD4 estimate varied according to covariate specification and the subset of patients with available mutation data.

These observations do not contradict the biological tumor-suppressive role of PDCD4 but indicate that bulk-tumor PDCD4 mRNA expression cannot currently be regarded as an independently validated prognostic marker in LUAD. Previous studies have also shown that PDCD4 protein and mRNA levels may not correspond directly in lung tumors [20], emphasizing the need for protein-level and functional validation.

### 4.2. AVEN

AVEN is an anti-apoptotic protein that interacts with BCL-xL and APAF1 and inhibits caspase activation [11]. In the present analysis, AVEN showed the most statistically robust survival association among the five evaluated genes, with higher expression associated with poorer survival in TCGA LUAD after correction for multiple testing.

This finding is biologically compatible with the anti-apoptotic properties of AVEN and is directionally consistent with a previous TCGA-based LUAD analysis [21]. However, because of the overlap in data source, that study does not constitute independent replication of the present TCGA finding. In addition, high AVEN expression was associated with enrichment of several proliferative and metabolic programs, including MYC targets, mTORC1 signaling, DNA repair, oxidative phosphorylation, and unfolded protein response.

Nevertheless, these observations require cautious interpretation. The adjusted AVEN association in GSE30219 did not remain significant after FDR correction, and the GSEA findings were derived from bulk transcriptomic data. Thus, neither the survival association nor the pathway enrichment establishes direct oncogenic activity or clinical prognostic utility.

### 4.3. PDCD2, PDCD5, and PDCD10

PDCD2, PDCD5, and PDCD10 showed several nominal survival and co-expression associations, but most did not remain significant after multiple-testing correction.

PDCD2 participates in ribosome biogenesis [6], whereas PDCD5 has been linked to p53 regulation, genotoxic stress responses, lung-cancer susceptibility, and experimental tumor suppression [8,9,22,23]. These biological roles provide plausible contexts for tumor-associated expression changes, but transcript abundance alone cannot establish functional activity.

PDCD10 has been reported to have context-dependent cellular and cancer-related functions [10]. In the present analysis, higher PDCD10 expression showed a nominal adverse association in LUAD and a nominal favorable association in LUSC. The favorable LUSC association was also apparent in the secondary median-based analysis, but it did not remain significant in the primary continuous-expression model after FDR correction and was not independently reproduced in GSE30219. Consequently, the present findings do not establish a tumor-suppressive or oncogenic role for PDCD10 in either histological subtype.

### 4.4. Co-Expression and Histology-Specific Context

The co-expression analyses demonstrated that PDCD-related genes are not uniformly correlated and may reflect different transcriptional contexts in LUAD and LUSC. PDCD2, PDCD5, PDCD10, and AVEN showed several positive correlations, whereas PDCD4 demonstrated a comparatively distinct expression pattern.

However, the magnitude of several statistically significant correlations was small. Given the size of the TCGA cohorts, small correlation coefficients may reach statistical significance without representing strong biological relationships. Accordingly, the driver-gene analyses should be interpreted as transcript co-expression rather than evidence of direct functional interaction.

The observation that associations differed between LUAD and LUSC also does not by itself establish a formal gene-by-histology interaction. The present study was designed primarily for histology-stratified analyses, and subtype-specific differences should therefore be regarded as comparative patterns rather than proof of differential biological effect.

### 4.5. GSEA and Biological Interpretation

The GSEA findings identified several proliferative and metabolic pathways associated with target-gene expression groups. High AVEN expression in LUAD was associated with multiple growth- and metabolism-related Hallmark gene sets, whereas low PDCD4 expression showed several enrichment patterns in the legacy RSEM analysis.

However, PDCD4-associated pathway enrichment was not reproduced using STAR TPM expression. Differences in transcript quantification, gene annotation, and median-based group assignment can influence ranked gene lists and enrichment statistics.

Furthermore, GSEA was based on bulk-tumor expression and was not adjusted for tumor purity, immune-cell infiltration, stromal composition, or other clinical characteristics. Therefore, the enrichment results should be interpreted as pathway associations with expression states, rather than as evidence that individual PDCD-related genes directly regulate the identified pathways.

### 4.6. Strengths and Limitations

A major strength of this study is the parallel evaluation of five PDCD-related genes using a common analytical framework across LUAD and LUSC. The analytical framework incorporated continuous-expression Cox models, explicit multiple-testing correction, formal proportional-hazards diagnostics, multicollinearity assessment, nonlinear modeling, incremental prognostic evaluation, and clinically adjusted external analysis.

Several limitations remain.

First, the study is retrospective and based on publicly available datasets. Because several analyses were undertaken after the initial findings had been examined, the study should be regarded as exploratory rather than prospectively confirmatory.

Second, complete-case analysis may introduce selection bias, particularly in analyses requiring molecular covariates such as EGFR mutation status. Treatment information, performance status, comorbidities, recurrence outcomes, and some subtype-specific molecular alterations were not consistently available.

Third, GSE30219 included only 83 LUAD and 61 LUSC patients. Node-positive groups were particularly small, and the available external covariates differed from those used in TCGA. Differences between RNA sequencing and microarray platforms further limit direct comparison of absolute expression values.

Fourth, proportional-hazards diagnostics identified time-dependent behavior in some stage terms, although not in the primary target-gene terms. Multicollinearity diagnostics did not indicate severe inflation, but simultaneous-gene coefficients remain dependent on model specification.

Fifth, GSEA was not adjusted for tumor purity or immune/stromal composition. Bulk tumor expression therefore cannot distinguish tumor-cell-intrinsic expression from expression originating from nonmalignant cells in the tumor microenvironment.

Finally, no functional experiments, protein-level validation, prospective prognostic assessment, or treatment-response analyses were performed. Larger independent cohorts and cell-type-resolved and functional studies are needed before clinical or mechanistic conclusions can be established.

## 5. Conclusions

This study demonstrates that PDCD-related genes show heterogeneous clinical, molecular, and survival associations across LUAD and LUSC.

After continuous-expression modeling and multiple-testing correction, higher AVEN expression was associated with poorer overall survival in TCGA LUAD. In contrast, the favorable association of PDCD4 observed in the secondary median-based analysis was not significant in the primary adjusted analysis and did not demonstrate established incremental prognostic value beyond conventional clinical factors.

No adjusted survival association was independently reproduced after FDR correction in GSE30219.

Therefore, the present findings identify candidate histology-specific expression associations rather than validated prognostic biomarkers. Further validation in larger, clinically annotated independent cohorts, together with protein-level and functional studies, is required.

## Figures and Tables

**Table 1:** Baseline characteristics and analysis populations.

Characteristic	TCGA		GSE30219	
	LUAD	LUSC	LUAD	LUSC
Expression cohort	492	484	83	61
Survival eligible	490	483	83	61
Deaths with known event status	178	210	43	43
Primary model *n*/deaths	469/167	467/205	83/43	61/43
Age, median (IQR), years	66 (59–73)	68 (62–73)	60 (55–69)	63 (57–70)
Male, *n*/*N* (%)	224/490 (45.7%)	358/483 (74.1%)	65/83 (78.3%)	56/61 (91.8%)
Ever smoking, *n*/*N* (%)	408/478 (85.4%)	454/472 (96.2%)	Not available	Not available
Stage I, *n*/*N* (%)	262/482 (54.4%)	237/479 (49.5%)	Not available	Not available
Stage II, *n*/*N* (%)	117/482 (24.3%)	152/479 (31.7%)	Not available	Not available
Stage III, *n*/*N* (%)	79/482 (16.4%)	83/479 (17.3%)	Not available	Not available
Stage IV, *n*/*N* (%)	24/482 (5.0%)	7/479 (1.5%)	Not available	Not available
T1, *n*/*N* (%)	164/487 (33.7%)	109/483 (22.6%)	69/83 (83.1%)	49/61 (80.3%)
N0, *n*/*N* (%)	316/479 (66.0%)	308/477 (64.6%)	80/83 (96.4%)	52/61 (85.2%)
EGFR mutation, *n*/*N* (%)	29/211 (13.7%)	21/288 (7.3%)	Not available	Not available

Values are n/nonmissing denominator (%) unless otherwise specified. T1 and N0 are shown as selected baseline categories because these categories served as the reference levels for the grouped T- and N-stage variables in the external Cox models; complete T- and N-stage distributions are provided in Supplementary Table S1. Missing counts refer to the expression cohort. Death counts are among participants with known event status; survival eligibility additionally requires positive follow-up.

**Table 2:** Primary continuous-expression Cox analyses in TCGA.

Histology	Gene	*n*/Deaths	Adjusted HR (95% CI)	*p* Value	BH q
LUAD	*PDCD2*	469/167	1.092 (0.936–1.275)	0.261	0.327
LUAD	*PDCD4*	469/167	0.875 (0.749–1.021)	0.090	0.150
LUAD	*PDCD5*	469/167	1.186 (1.027–1.370)	0.020	0.067
LUAD	*PDCD10*	469/167	1.168 (1.006–1.355)	0.041	0.103
LUAD	*AVEN*	469/167	1.272 (1.091–1.483)	0.002	0.022
LUSC	*PDCD2*	467/205	0.946 (0.810–1.104)	0.479	0.479
LUSC	*PDCD4*	467/205	0.939 (0.816–1.080)	0.377	0.419
LUSC	*PDCD5*	467/205	0.879 (0.758–1.020)	0.088	0.150
LUSC	*PDCD10*	467/205	0.843 (0.733–0.969)	0.016	0.067
LUSC	*AVEN*	467/205	0.919 (0.801–1.055)	0.229	0.327

HRs represent a one-SD increase in log2 expression. Each gene was fitted separately with adjustment for age per 10 years, sex, corrected smoking history, and categorical pathologic stage. Benjamini–Hochberg q values cover all ten gene–histology tests. Unadjusted and secondary models are provided in Supplementary Table S4.

**Table 3:** Exploratory incremental assessment of *PDCD4* in TCGA LUAD.

Measure	Clinical Variables	Clinical Variables + *PDCD4*
Patients/deaths	469/167	469/167
Fitted coefficients	6	7
Apparent C-index	0.667	0.680
Bootstrap 95% CI for apparent C	0.627–0.722	0.640–0.729
Estimated optimism	0.015	0.017
Optimism-corrected C-index	0.651	0.663
Change in apparent C-index	Reference	0.014 (−0.004 to 0.031)
Change in optimism-corrected C	Reference	0.012
Likelihood-ratio comparison	Reference	χ^2^ = 2.868; df = 1; *p* = 0.090

Both models were fitted using identical complete cases. Clinical variables were age, sex, corrected smoking history, and categorical pathologic stage. Intervals are percentile bootstrap intervals from 1000 valid patient resamples. The paired C-index difference interval is not an interval for the optimism-corrected difference. The likelihood-ratio comparison was exploratory and was not adjusted for multiplicity.

**Table 4:** Continuous-expression external survival analyses in GSE30219.

**LUAD (83 patients; 43 deaths)**						
**Gene**	**Unadjusted HR (95% CI)**	***p* value**	**BH q**	**Adjusted HR (95% CI)**	***p* value**	**BH q**
PDCD2	1.459 (1.045–2.038)	0.027	0.089	1.481 (1.023–2.145)	0.037	0.187
PDCD4	0.878 (0.663–1.161)	0.360	0.600	0.837 (0.637–1.098)	0.199	0.332
PDCD5	1.226 (0.910–1.652)	0.180	0.359	1.291 (0.909–1.833)	0.154	0.308
PDCD10	1.432 (1.061–1.931)	0.019	0.089	1.518 (1.105–2.087)	0.010	0.100
AVEN	1.460 (1.048–2.033)	0.025	0.089	1.356 (0.961–1.912)	0.083	0.276
**LUSC (61 patients; 43 deaths)**						
**Gene**	**Unadjusted HR (95% CI)**	***p* value**	**BH q**	**Adjusted HR (95% CI)**	***p* value**	**BH q**
PDCD2	0.963 (0.714–1.300)	0.807	0.948	0.983 (0.717–1.347)	0.914	0.961
PDCD4	0.971 (0.707–1.332)	0.853	0.948	0.921 (0.675–1.257)	0.604	0.863
PDCD5	1.003 (0.726–1.385)	0.987	0.987	1.035 (0.749–1.430)	0.836	0.961
PDCD10	0.826 (0.625–1.092)	0.179	0.359	0.779 (0.564–1.075)	0.129	0.308
AVEN	1.030 (0.762–1.393)	0.847	0.948	1.007 (0.753–1.347)	0.961	0.961

HRs represent a one-SD increase in log2 probe expression. Adjusted models include age per 10 years, sex, T1 versus T2–T4, and N0 versus N1–N3. Benjamini–Hochberg correction covers ten gene–histology tests separately for adjusted and unadjusted model families. The available clinical covariates differ from those in TCGA, and node-positive groups were small.

## Data Availability

The datasets analyzed in this study are publicly available. Transcriptomic and clinicopathological data for lung adenocarcinoma (LUAD) and lung squamous cell carcinoma (LUSC) were obtained from The Cancer Genome Atlas (TCGA), with primary-tumor expression profiles accessed through UCSC Xena. The external cohort GSE30219, generated using the Affymetrix Human Genome U133 Plus 2.0 Array platform (GPL570), is publicly available from the Gene Expression Omnibus (GEO). Hallmark gene sets used for gene set enrichment analysis were obtained from the Molecular Signatures Database (MSigDB). Detailed statistical outputs and supporting analyses generated in this study are provided in the Supplementary Materials. Further information regarding the analyses is available from the corresponding author.

## Supplementary Materials

The following supporting information can be downloaded at https://www.mdpi.com/article/10.3390/genes17101238/s1. Table S1: Cohort assembly, baseline characteristics, follow-up, and clinical-data audit; Table S2: Clinicopathological associations of PDCD-related gene-expression groups in TCGA LUAD and LUSC; Table S3: Pearson and Spearman co-expression analyses among PDCD-related genes and histology-relevant oncogenic-driver transcripts; Table S4: Complete primary, secondary, and sensitivity Cox regression analyses; Table S5: Proportional-hazards diagnostics, multicollinearity assessment, nonlinearity analyses, and detailed PDCD4 incremental-performance analyses; Table S6: GSE30219 probe, expression, median-group, Kaplan–Meier, and sensitivity-analysis audit; Table S7: Complete Hallmark GSEA results; Figure S1: TCGA median-based Kaplan–Meier curves for all five genes in LUAD and LUSC; Figure S2: GSE30219 median-based Kaplan–Meier curves for all five genes in LUAD and LUSC; Figure S3: Hallmark GSEA using STAR TPM as an expression-resource sensitivity analysis; Figure S4: Adjusted PDCD4 natural cubic-spline analyses in TCGA and GSE30219; Figure S5: Selected scaled Schoenfeld residual diagnostic plots for the LUAD PDCD4 and AVEN primary models.
