# Supplementary material for: Histology-Stratified Clinical and Prognostic Associations of PDCD-Related Genes in Lung Adenocarcinoma and Lung Squamous Cell Carcinoma
Source: Genes (Basel). 2026 Oct 8;17(10):1238. doi: 10.3390/genes17101238 (PMC13704477; doi:10.3390/genes17101238)

Supplementary Figure S1. TCGA median-based Kaplan–Meier analyses.

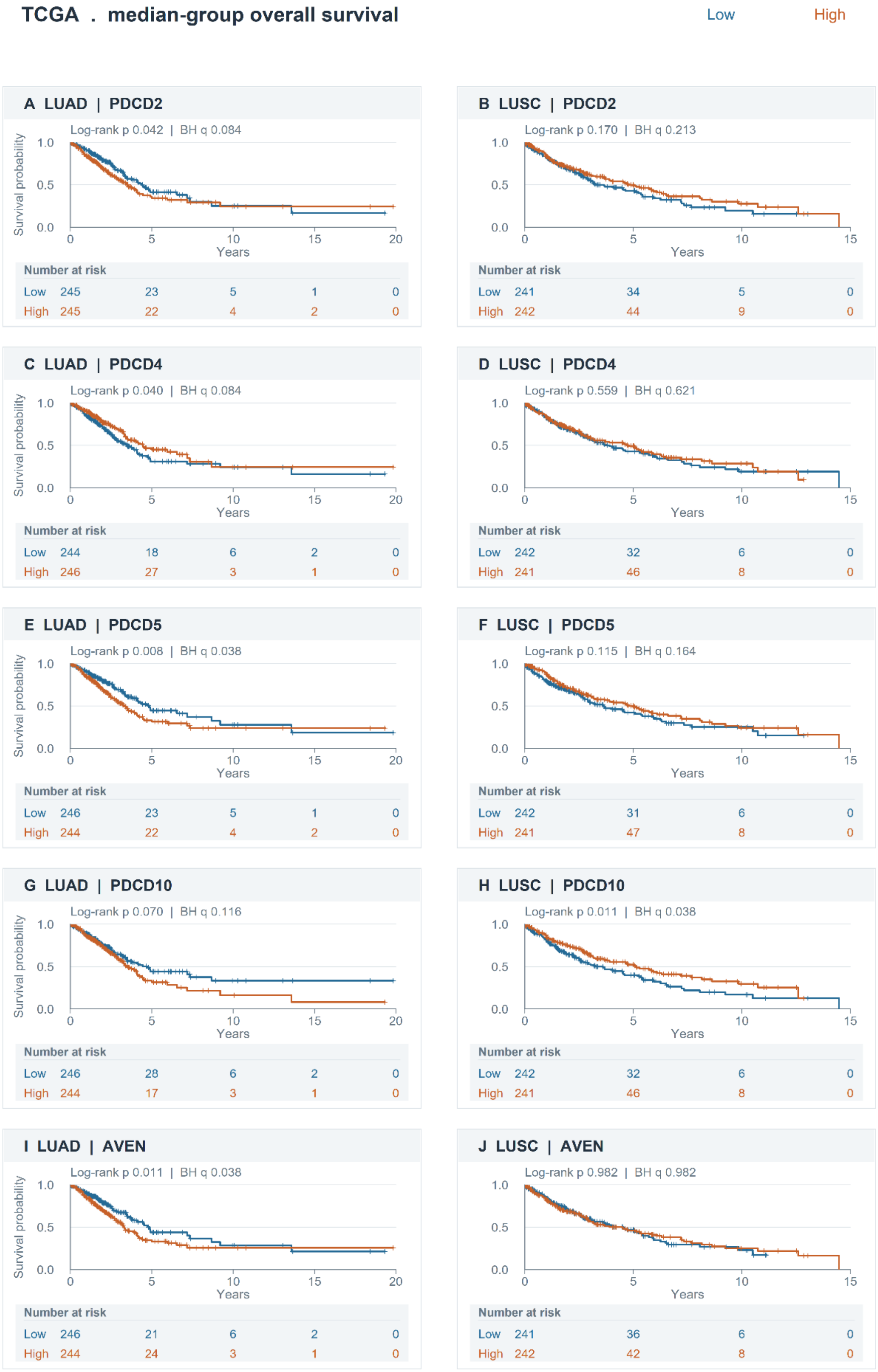

Histology-specific medians; censoring marked +. All ten comparisons are displayed.

Supplementary Figure S2. GSE30219 median-based Kaplan–Meier analyses.

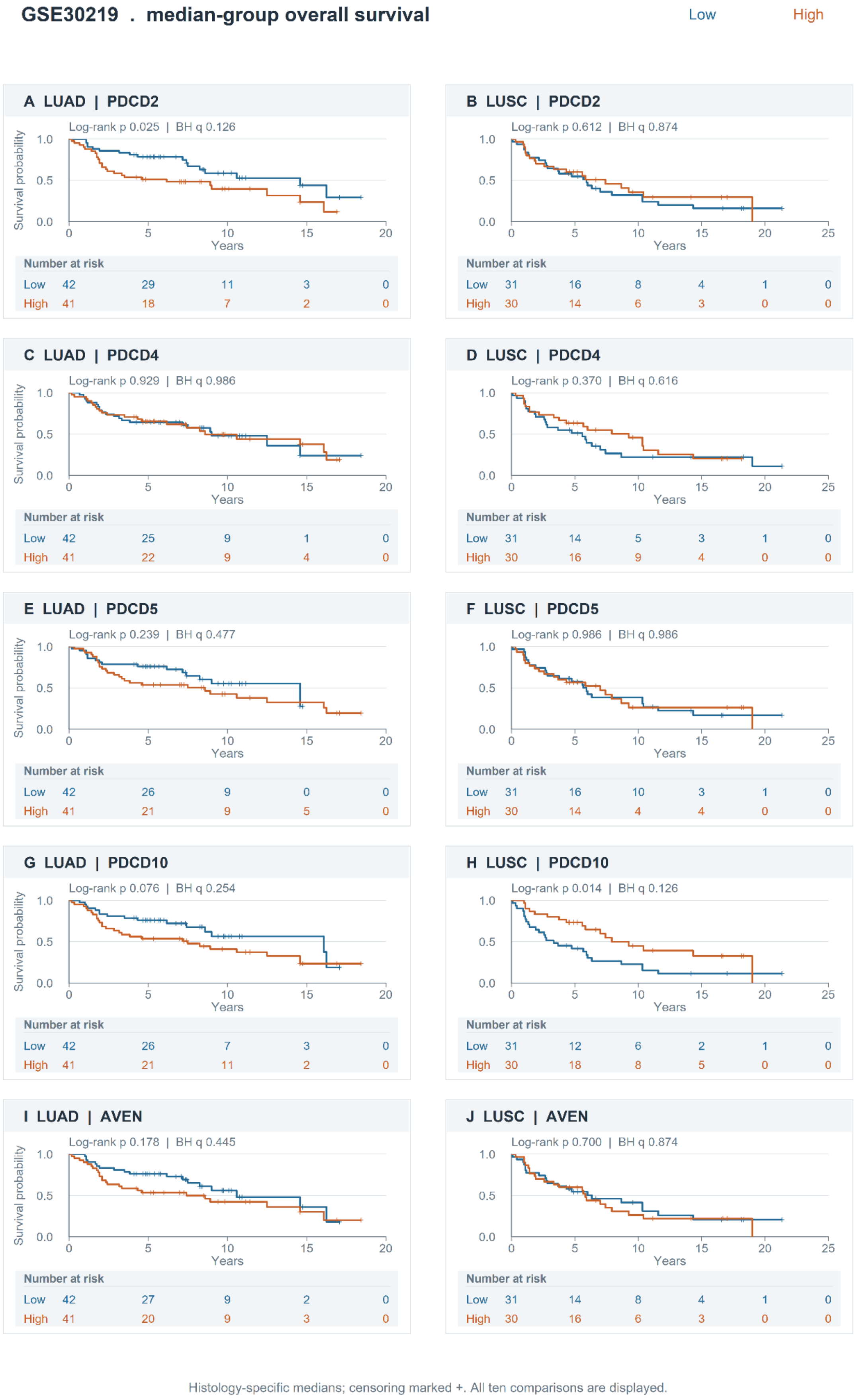

Supplementary Figure S3. Hallmark GSEA using STAR TPM expression.

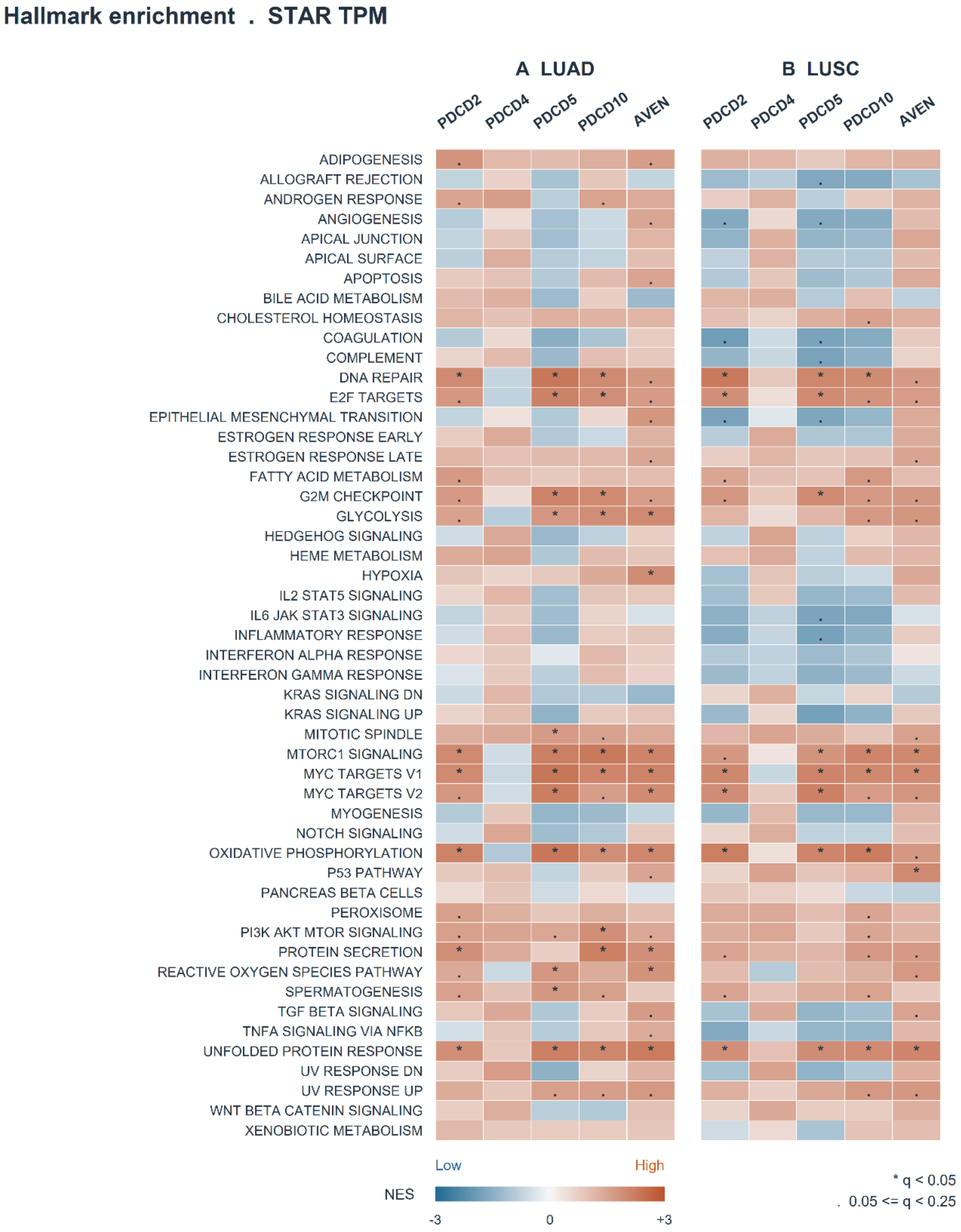

Supplementary Figure S4. Adjusted PDCD4 natural cubic-spline analyses in TCGA and GSE30219.

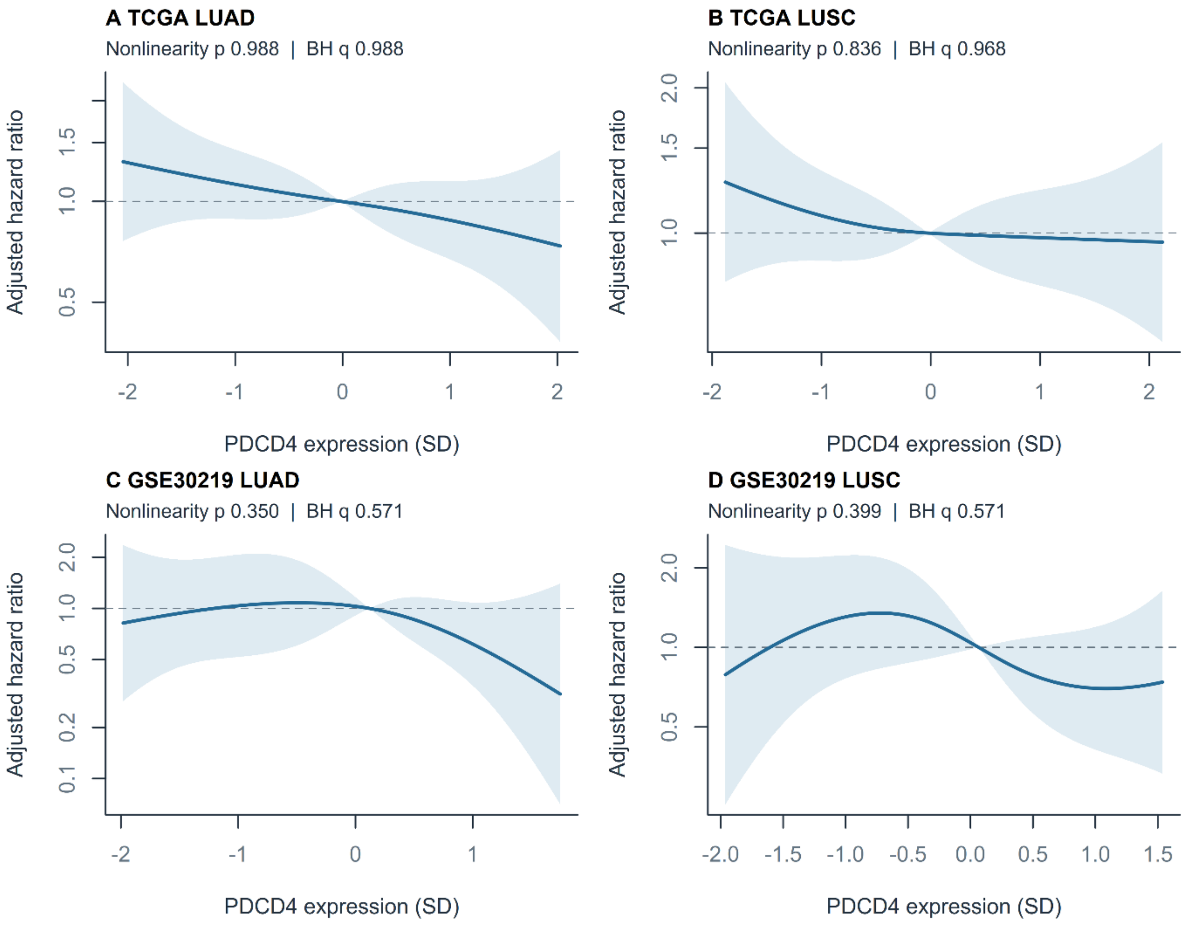

Supplementary Figure S5. Scaled Schoenfeld residual diagnostics for the LUAD PDCD4 and AVEN primary models.

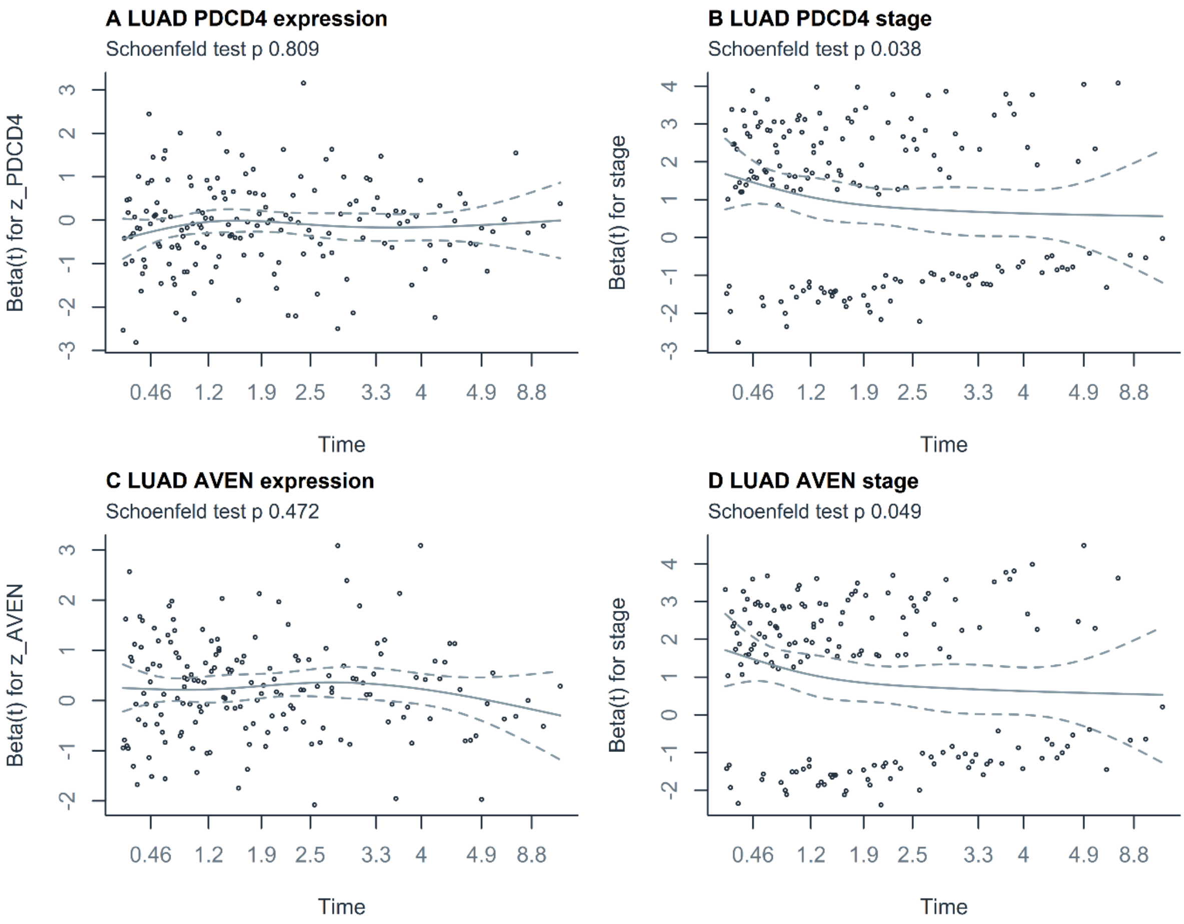

Supplement: Supplementary file 1 [file genes-17-01238-s001.zip › genes-4547976-figures.pdf]
